# Predictive value of characteristics of resected parathyroid glands for persistent secondary hyperparathyroidism during parathyroidectomy

**DOI:** 10.1186/s12893-023-01936-5

**Published:** 2023-02-14

**Authors:** Yaoyu Huang, Jing Wang, Ming Zeng, Huiting Wan, Ningning Wang, Guang Yang

**Affiliations:** Department of Nephrology, The First Affiliated Hospital of Nanjing Medical University, Jiangsu Province Hospital, Nanjing Medical University, 300 Guangzhou Road, Nanjing, 210029 Jiangsu China

**Keywords:** Secondary hyperparathyroidism, Parathyroidectomy, Minimum weight, Persistent secondary hyperparathyroidism

## Abstract

**Background:**

Parathyroidectomy (PTX), an effective treatment for refractory secondary hyperparathyroidism (SHPT) in chronic kidney disease (CKD) patients, still has a high persistent rate. This study aimed to analyze the predictive value of characteristics of resected parathyroid glands for postoperative persistent SHPT.

**Methods:**

The clinical data of patients with persistent SHPT and successful PTX controls who had or underwent total parathyroidectomy with forearm autotransplantation (TPTX + AT) was retrospectively collected. The characteristics including the number, minimum weight, maximum weight and total weight of resected parathyroid glands from each patient were recorded. Characteristics and cutoff value of resected parathyroid glands for the prediction of persistent SHPT were analyzed.

**Results:**

A total of 227 patients (62 persistent SHPT patients and 165 successful PTX controls) were enrolled in the study. Forty-one (66%) persistent SHPT cases related to supernumerary parathyroid and the remaining 21 (34%) cases related to residual undetected parathyroid. In addition, ectopic parathyroid was found in 8 patients (13%) before PTX. The average number of resected glands in the persistent SHPT group and successful PTX group was 3.53 ± 0.72 and 3.93 ± 0.25 respectively (*p* < 0.001). There was significance in the number of patients with different resected parathyroid glands between two groups (*p* < 0.001). When the resected gland number was 4, minimum weight of the parathyroid was noted to be heavier in the persistent SHPT group than that in the successful PTX group (0.52 ± 0.31 g vs. 0.38 ± 0.19 g, *p* < 0.001). For persistent SHPT prediction, cutoff value of minimum weight was 0.535 g, with sensitivity of 46% and specificity of 82% (AUC = 0.611; *p* = 0.029).

**Conclusions:**

Major reason for the persistent SHPT is the existence of supernumerary parathyroid glands or resection of less than 4 glands. When 4 glands were resected, a minimum total parathyroid gland weight heavier than 0.535 g implied the potential presence of a missed supernumerary parathyroid gland, which also contributed to the persistent SHPT.

## Background

Secondary hyperparathyroidism (SHPT) is a common and severe complication of chronic kidney disease (CKD) that can result in a broad spectrum of mineral metabolism disorders, accelerated atherosclerosis and serious cardiovascular events [1]. In spite of recent advances in therapy, medical treatment failure still occurs in a significant number of patients in whom surgical treatment is indicated [2]. Parathyroidectomy (PTX) is associated with a reduction in both all-cause and cardiovascular mortality, and improves symptoms that affect quality of life [3, 4].

At present, the main surgical options include subtotal parathyroidectomy (SPTX), total parathyroidectomy (TPTX) and total parathyroidectomy with forearm autotransplantation (TPTX + AT). All the three surgical options are performed in China, However, there is ongoing debate about optimal surgical treatment for SHPT. Compared to TPTX with or without autotransplantation, SPTX may be associated with a higher rate of persistence and recurrence, and more potential risk of second cervical surgery [5–7]. Thus, many surgeons consider TPTX with or without autotransplantation as a favored approach. However, it is sometimes difficult to resect all parathyroid glands because of the existence of supernumerary or ectopic parathyroids. The rate of persistent SHPT after TPTX with or without autotransplantation is still up to 0.4–25% [8, 9].

This study investigates the difference of resected parathyroid glands between the successful PTX group and the persistent SHPT group in patients undergoing TPTX + AT. The predictive value of characteristics of resected parathyroid glands for persistent SHPT during PTX is also analyzed.

## Methods

### Study population

From April 2013 to November 2019, persistent SHPT patients hospitalized in the First Affiliated Hospital of Nanjing Medical University were enrolled and successful PTX patients were matched as controls, who hospitalized at the same time with similar dialysis age. All patients had or underwent TPTX + AT during hospitalization. This study has been approved by the Ethics Committee of the First Affiliated Hospital of Nanjing Medical University. Informed consent was obtained from all patients.

PTX was performed in refractory SHPT patients who failed to respond to medical therapy [10]. The surgical indications included: (1) persistent serum intact parathyroid hormone (iPTH) > 800 pg/mL; (2) hypercalcemia and/or hyperphosphatemia that could not be controlled by medical therapy; (3) obvious clinical manifestations such as bone pain, pruritus, ectopic calcification or fracture; and (4) and at least one enlarged parathyroid gland with a diameter greater than 1 cm discovered by ultrasound or a radiopharmaceutical technetium-99 m-methoxyisobutylisonitrile (99mTc-MIBI) scan [8, 10].

### Surgical procedure

Preoperative evaluations included thyroid/parathyroid ultrasonography and parathyroid scintigraphy (99mTc-MIBI) for identifying the number, size, and location of parathyroid glands. TPTX + AT without thymectomy was performed routinely under general anesthesia in all SHPT patients. All operations were performed by the same surgeon. Bilateral neck exploration was routinely performed to make sure all detected parathyroid glands including supernumerary and ectopic parathyroids would be resected.

Intraoperative fast frozen section analysis was routinely adopted to verify that the resected specimen was benign hyperplasia parathyroid tissue. The selected diffuse hyperplasia parathyroid fragment was cut into slices of about 1 mm^3^. Then, eight slices were transplanted into forearm muscles without an arteriovenous fistula for hemodialysis. After surgery, pathological sections were sent to department of pathology for conventional pathology inspection [8].

### Observation parameters

Routine blood tests, serum iPTH levels and biochemical indices including serum creatinine, urea nitrogen, albumin, calcium, phosphorus and alkaline phosphatase were measured. The recommended range of serum iPTH was 12–88 pg/mL among healthy people.

The characteristics including the number and total weight of resected parathyroid glands, and the minimum and maximum weight per resected gland from each patient in PTX were recorded during the operation.

Corrected calcium (cCa) was calculated with the following formula: [cCa (mmol/L) = total calcium (mmol/L) + (40—Albumin (g/L)) * 0.025 (mmol/L)].

Hyperplastic parathyroid glands located inside the superior mediastinum and thyroid were regarded as ectopic parathyroid glands [11], and a supernumerary gland case was defined as more than four parathyroid glands in a patient.

### Definition of successful PTX and persistent SHPT

Patients with peak serum iPTH < 50 pg/mL at the first postoperative week were defined as the successful PTX group directly. Then, patients with peak serum iPTH > 50 pg/mL at the first postoperative week were followed up and received iPTH testing every month to verify the operative outcomes. Depending on serum iPTH values within 6 months, patients with peak iPTH < 300 pg/mL were also classified as the successful PTX group, and those whose peak iPTH were > 300 pg/mL or who required PTH-lowering therapy were regarded as persistent SHPT [8, 12].

The numbers of resected glands in both groups were recorded. Minimum weight, maximum weight and total weight of resected parathyroid glands in the persistent SHPT group were compared to that in the successful PTX group respectively according to the resected gland number.

### Statistical analysis

The data were analyzed by using the Statistical Package for the Social Sciences (SPSS) version 22.0. Continuous variables were expressed as mean ± standard deviation (SD) or median (interquartile range). Categorical variables were presented as number and proportion. Comparisons between groups were performed using an independent samples *t* or Wilcoxon rank sum test for continuous variables and a chi-squared or Fisher’s exact test for categorical variables. Significance was defined as *p* < 0.05. Receiver operating characteristic (ROC) curves were used to identify the cutoff value of the minimum weight of resected parathyroid glands for prediction of persistent SHPT. Diagnostic accuracy was expressed through sensitivity, specificity, and the area under the ROC curve (AUC).

## Results

### Participant characteristics before and after surgery

A total of 227 patients were enrolled in the study. Sixty-two persistent SHPT patients (35 males) and 165 successful PTX controls (100 males) after surgery were assigned as the persistent SHPT group and the successful PTX group (Table 1). All patients received regular hemodialysis three times a week or daily peritoneal dialysis (211 hemodialysis cases and 16 peritoneal dialysis cases). There were no differences in baseline characteristics between the two groups.

**Table 1:** Baseline demographics, clinical characteristics and laboratory results in successful and persistent subgroups of SHPT patients

Clinical characteristics	Successful PTX (*n* = 165)	Persistent SHPT (*n* = 62)
*Demographics*		
Age (y)	46.74 ± 10.33	45.39 ± 10.69
Gender (male/female)	100/65	35/27
*Dialysis mode, n (%)*		
Hemodialysis	153 (93%)	58 (94%)
Peritoneal dialysis	12 (7%)	4 (6%)
*Dialysis vintage(months)*	92.27 ± 41.17	95.56 ± 47.10
*Laboratory values*		
Hemoglobin (g/L)	101.70 ± 20.34	106.10 ± 19.12
Creatinine (umol/L)	915.70 ± 262.49	945.38 ± 263.99
Urea (mmol/L)	23.61 ± 7.12	24.87 ± 8.25
Albumin (g/L)	38.34 ± 3.63	38.42 ± 3.73
cCa (mmol/L)	2.62 ± 0.26	2.59 ± 0.21
Phosphorus (mmol/L)	2.18 ± 0.57	2.18 ± 0.52
ALP (U/L)	311.8 (161.0, 768.7)	353.9 (149.6, 671.9)
ln ALP	5.84 ± 0.97	5.80 ± 0.92
iPTH (pg/ml)	1971.8 (1508.3, 2670.2)	2237.7 (1551.3, 3092.5)
ln iPTH	7.53 ± 0.48	7.60 ± 0.49

None of the differences between two groups is significant (*p* > 0.05)

Data are presented as mean ± SD, numbers, percentages and quartile, as appropriate

SHPT, secondary hyperparathyroidism; cCa, corrected calcium; ALP, alkaline phosphatase; iPTH, intact parathyroid hormone; ln, the natural logarithm

Compared to persistent SHPT group, iPTH and cCa were lower in successful PTX group 6 months after the operation, as shown in Table 2. While no significant differences were identified in albumin, phosphorus and alkaline phosphatase (ALP) during the follow-up period (Table 2).

**Table 2:** Follow-up laboratory parameters 6 months after the operation

Clinical characteristics	Successful PTX (*n* = 165)	Persistent SHPT (*n* = 62)	*P* value
Albumin (g/L)	38.56 ± 4.19	38.03 ± 4.05	0.397
cCa (mmol/L)	2.52 ± 0.24	2.61 ± 0.23	0.011
Phosphorus (mmol/L)	2.20 ± 0.45	2.09 ± 0.54	0.134
ALP (U/L)	291.0 (162.1, 496.6)	330.0 (146.4, 536.7)	-
ln ALP	5.67 ± 0.77	5.76 ± 0.81	0.454
iPTH (pg/ml)	85.3 (60.8, 137.3)	855.4 (567.2, 1367.5)	-
ln iPTH	4.50 ± 0.56	6.76 ± 0.63	< 0.001

Data are presented as mean ± SD and quartile, as appropriate

cCa, corrected calcium; ALP, alkaline phosphatase; iPTH, intact parathyroid hormone; ln, the natural logarithm

### Causes for persistent SHPT

A total of 62 cases showed persistent SHPT after surgery: forty-one (66%) related to supernumerary parathyroid (the number of resected parathyroid is 4) and the remaining 21 (34%) related to residual undetected parathyroid (the number of resected parathyroid is fewer than 4). In addition, ectopic parathyroid was found in 8 patients (13%) by parathyroid ultrasonography or parathyroid scintigraphy (99mTc-MIBI) before the PTX (Table 3).

**Table 3:** Preoperative imaging and characteristics of detected and resected parathyroid glands

Variables	Successful PTX (*n* = 165)	Persistent SHPT (*n* = 62)	*P* value
*Preoperative imaging*			
Ectopic parathyroid gland, *n* (%)	7 (4%)	8 (13%)	0.041
*Patients with different resected parathyroid glands*			
Number = 2	0	8	< 0.001
Number = 3	11	13	
Number = 4	154	41	
*Characteristics of resected parathyroid glands*			
Average number of resected parathyroid glands	3.93 ± 0.25	3.53 ± 0.72	< 0.001
Number of resected parathyroid glands	4 (4, 4)	4 (3, 4)	-
Number = 2	*n* = 0	*n* = 8	
Minimum weight (g)	-	0.65 (0.16, 1.48)	-
Maximum weight (g)	-	1.00 (0.80, 2.05)	-
Total weight (g)	-	1.60 (1.33, 3.25)	-
Number = 3	*n* = 11	*n* = 13	
Minimum weight (g)	0.35 ± 0.14	0.42 ± 0.26	0.407
Maximum weight (g)	1.85 ± 0.97	1.48 ± 0.67	0.282
Total weight (g)	2.99 ± 1.36	2.65 ± 1.16	0.522
Number = 4	*n* = 154	*n* = 41	
Minimum weight (g)	0.38 ± 0.19	0.52 ± 0.31	0.009
Maximum weight (g)	1.80 ± 0.86	1.84 ± 1.10	0.836
Total weight (g)	3.93 ± 1.62	4.24 ± 2.26	0.421

Data are presented as mean ± SD, numbers, percentages and quartile, as appropriate

### Characteristics of resected parathyroid glands

**Fig. 1:**
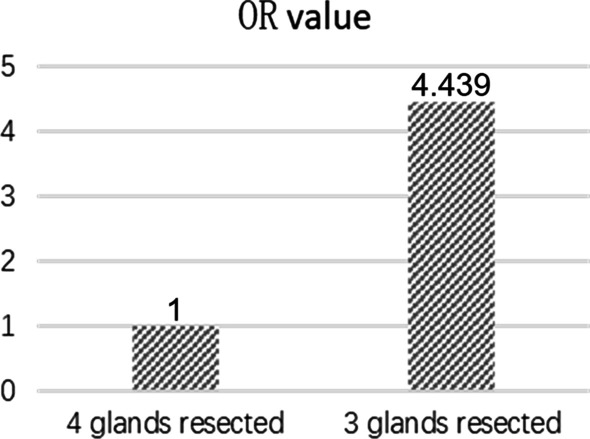
OR value of the number of resected glands for persistent SHPT

The average number of resected glands was 3.53 ± 0.72 and 3.93 ± 0.25 in the persistent SHPT group successful PTX group (*p* < 0.001) (Table 3). There was significant difference in the number of patients with different resected parathyroid glands between two groups (*p* < 0.001). The number of removed glands fewer than 4 might increased the risk of persistent SHPT. Compared to “4 glands” removed patients, “3 glands” resected patients had a higher risk of persistent SHPT with a 4.439 OR value (95% CI 1.853–10.635; *p* = 0.002) (Fig. 1), moreover, “2 glands” resected patients all suffered from persistent SHPT during follow up. Considering no data existed in the successful PTX group, the characteristics were not analyzed when the resected gland number was 2. There were no significant differences in minimum weight, maximum weight and total weight between the two groups when the resected gland number was 3. When the resected gland number was 4, minimum weight was noted to be heavier in the persistent SHPT group than that in the successful PTX group (0.52 ± 0.31 g vs. 0.38 ± 0.19 g, *p* < 0.001), while no significant differences were found in maximum weight and total weight (Table 2).

### Minimum weight cutoff value for the prediction of persistent SHPT

**Fig. 2:**
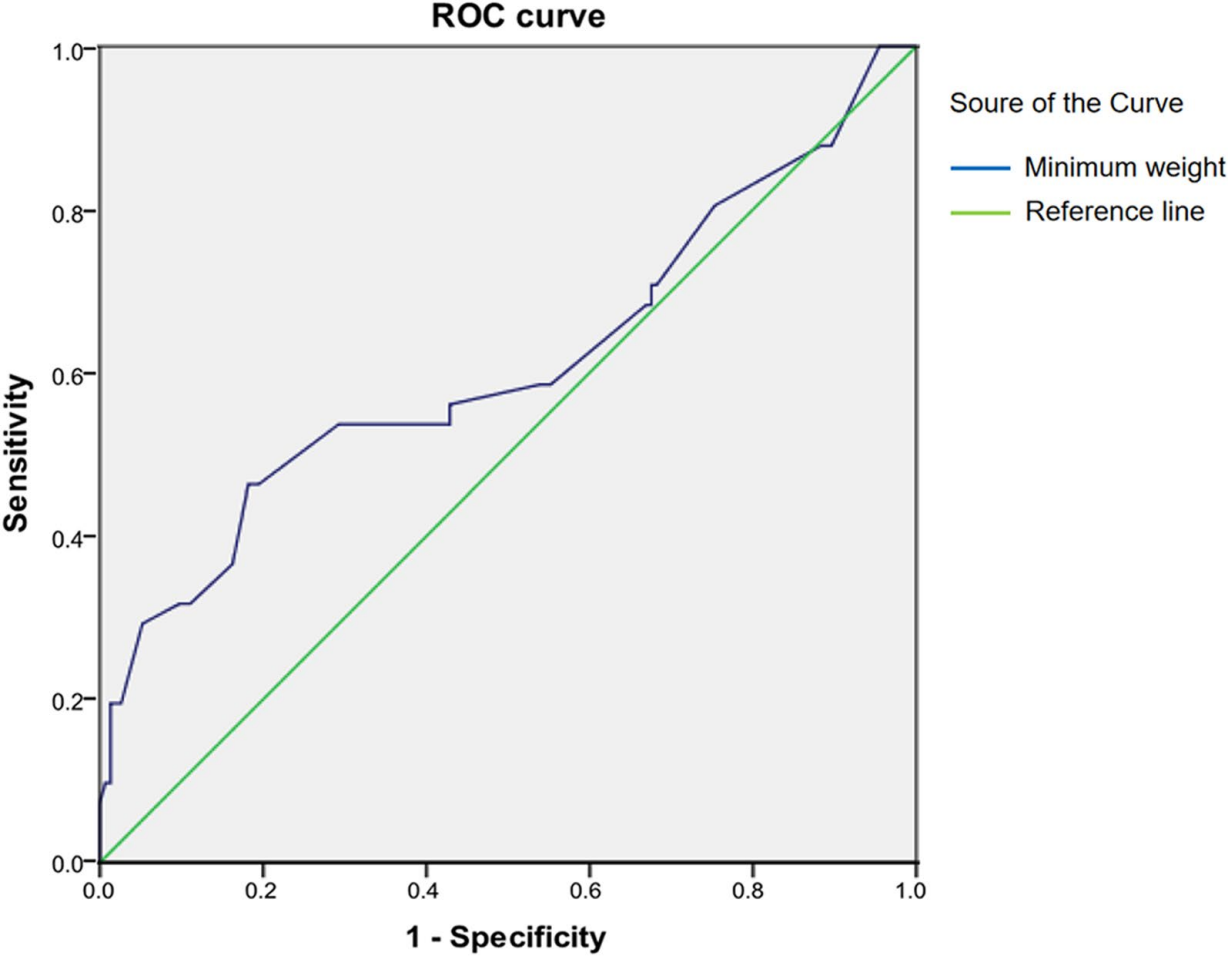
ROC curves of the minimum weight of the 4 resected glands for predicting of persistent SHPT

For persistent SHPT prediction, the cutoff value of minimum weight of 4 resected glands was 0.535 g, with a sensitivity of 46% and specificity of 82% (Fig. 2, AUC = 0.611; 95% CI 0.5–0.722; *p* = 0.029).

## Discussion

It was reported that about 10% of patients received PTX 10 years after dialysis and 30% of SHPT patients required operative treatment when the dialysis lasted for more than 20 years in Japan [13]. To achieve successful PTX, all parathyroid glands must be removed completely. However, variations in the number and location of the parathyroid glands make it difficult to perform successful PTX. Worse still, patients with SHPT tend to have a higher risk of supernumerary parathyroid glands [14, 15]. Shasha reviewed the quantity of excised glands in each SHPT patient and found that 87.85% of cases had 4 glands removed, 6.45% of cases had fewer than 4 glands removed, and 5.36% of cases had 5 or more glands removed (including one patient who had 11 glands) [16].

When persistent SHPT occurs after PTX, there must be residual parathyroid gland in the body. It could be associated with the following 2 situations: when the number of resected parathyroid is 4, surgeons may not continue to explore the operative region or not take too long to search the potential supernumerary parathyroid gland; when the number is fewer than 4, ectopic parathyroid exists or the residual gland is too small to detect. Our study summarized 62 cases showed persistent SHPT after surgery and found that the reasons for failure are due to supernumerary gland in 66% of cases, in 34% to unrecognized gland and 13% of cases were detected ectopic glands before the PTX, which was not similar to previous report. Alida et al. reported that unrecognized glands accounted for 70% of the failed cases, ectopic glands for 15% and supernumerary glands for 15% [17]. This difference may be attributed to the experience of surgeons.

The literatures and our previous study have demonstrated that intraoperative iPTH monitoring was a useful method for predicting successful PTX in SHPT patients [8, 18, 19], however, the waiting time for iPTH examining limits its application. In this study, by analyzing the characteristics of resected parathyroid glands, we found that the minimum weight of resected parathyroid glands was heavier in the persistent SHPT group than that in the successful PTX group when the resected gland number was 4 and the cutoff value was 0.535 g (sensitivity 46%, specificity 82%). The possible explanation was that some smaller parathyroid glands were potentially existent and difficult to find in operations. Although the sensitivity of the minimum weight predicting for persistent SHPT was not ideal, it was suggested that there may be a missed supernumerary parathyroid gland left when 4 parathyroids were detected along with minimum parathyroid weight was heavier than 0.535 g. In this situation, more careful examination of the surgical site for supernumerary or ectopic parathyroid was required to avoid persistent SHPT.

To the best of our knowledge, the characteristics of parathyroid excision and the minimum weight of resected glands to assess the risk of persistent SHPT have not been reported yet. Our study provides an easy distinguished option of for surgeon to realize the possible failure of PTX surgery. However, our study has some limitations. Firstly, as the procedure of operative exploration was closely related to the experience of surgeons, general applicability of our results needs further confirmation by prospective multi-center clinical research. Secondly, our sample size was small and control patients were matched according to age, sex and dialysis age. It is necessary to design a large sample study to further verify the predictive value of our results.

## Conclusions

In summary, our study reveals that major reason for the persistent SHPT is the existence of supernumerary parathyroid glands or resection of less than 4 glands. When 4 glands were resected, a minimum total parathyroid gland weight heavier than 0.535 g implied the potential presence of a missed supernumerary parathyroid gland, which also contributed to the persistent SHPT. In this event, more careful examination of the surgical site for supernumerary or ectopic parathyroid was required to avoid persistent SHPT.

## Data Availability

The data used and analyzed during the current study are available from the corresponding author upon reasonable request.

## Abbreviations

PTX: Parathyroidectomy
SHPT: Secondary hyperparathyroidism
CKD: Chronic kidney disease
TPTX: Total parathyroidectomy
TPTX + AT: Total parathyroidectomy with forearm autotransplantation
iPTH: Intact parathyroid hormone
99mTc-MIBI: Technetium-99 m-methoxyisobutylisonitrile
cCa: Corrected calcium
SPSS: Statistical package for the social sciences
SD: Standard deviation
ROC: Receiver operating characteristic
AUC: Area under the ROC curve
ALP: Alkaline phosphatase
